# Risk factors associated with *Cryptosporidia, Eimeria*, and diarrhea in smallholder dairy farms in Mukurwe-ini Sub-County, Nyeri County, Kenya

**DOI:** 10.14202/vetworld.2016.811-819

**Published:** 2016-08-05

**Authors:** S. G. Peter, George K. Gitau, S. Richards, J. A. Vanleeuwen, F. Uehlinger, C. M. Mulei, R. R. Kibet

**Affiliations:** 1 Department of Clinical Studies, Faculty of Veterinary Medicine, University of Nairobi, P.O. Box 29053-00625, Kangemi, Kenya; 2 Department of Health Management, Atlantic Veterinary College, University of Prince Edward Island, 550 University Avenue, Charlottetown, PEI C1A 4P3, Canada; 3 Department of Large Animal Clinical Sciences, Western College of Veterinary Medicine, University of Saskatchewan, 52 Campus Drive, Saskatoon SK S7N 5B4, Canada; 4 Department of Public Health, Pharmacology and Toxicology, Faculty of Veterinary Medicine, University of Nairobi, P.O. Box 29053-00625, Kangemi, Kenya

**Keywords:** *Cryptosporidium*, diarrhea incidence, *Eimeria*, neonatal dairy calves, risk factors

## Abstract

**Aim:**

This study was undertaken to determine the household, calf management, and calf factors associated with the occurrence of *Eimeria*, *Cryptosporidia*, and diarrhea in pre-weaned calves reared in smallholder dairy farms in Mukurwe-ini Sub-County of Nyeri County, Kenya. In addition, the study also evaluated factors associated with average daily weight gain in the same pre-weaned calves.

**Materials and Methods:**

A total of 112 newborn calves (63 males and 49 females) on 111 farms (1 set of twins) were followed for 2 months between June 2013 and August 2013. Two calves were lost to follow-up. A pre-tested questionnaire was used to collect data on household characteristics and calf management practices in the 111 selected farms. On the first visit to the farm (within 7 days of the birth of the calf), blood samples were collected from the jugular vein to assess the level of maternal immunity acquired by the calf, by determining the serum total protein and selenium concentration. At 4 and 6 weeks of age, fecal samples from the calves were collected to assess the presence of *Cryptosporidia* and *Eimeria* oocysts. Every 2 weeks for 2 months, the calves and their environments were examined, their 2-week consumption and health history were recorded, and weights were estimated with a weight tape. Each of the factors was evaluated in a univariable regression model and only those found to be significant (p≤0.20) were included in a multivariable model. Elimination of non-significant factors was done in the multivariable model through a backward elimination procedure so that only those variables which were confounders, and/or significant at (p≤0.05) remained in the final model.

**Results:**

About 37% (41/110) of the calves experienced diarrhea at least once during the 2-month study period. The overall period prevalence of *Eimeria* and *Cryptosporidia* was 42.7% (47/110) and 13.6% (15/110), respectively. Low serum protein was associated with 1.8 and 2.4 times the odds of *Eimeria* and *Cryptosporidia* infections, respectively. Lack of supervision of calf birth and low serum total protein were both associated with 1.3 times the odds of diarrhea incidence. Dirty calf pens, feeding <5 L of milk/day, and infection with *Eimeria* were associated with 0.105, 0.087, and 0.059 kg, respectively, reduced average daily weight gain of the calves.

**Conclusion:**

In the Kenyan context, calf diarrhea risk could be reduced through better supervision of parturition and colostrum provision. Specifically, the risk of *Eimeria* and *Cryptosporidia* infections could be reduced by optimizing the passive transfer of immunity to the newborn calves. Average weight gains of calves could be improved by good colostrum provision, pen hygiene, and preventing *Eimeria* infections.

## Introduction

Gastrointestinal parasites of ruminants are ubiquitous in the farm environment (parasitic eggs, larva, and cysts are present in the soil) and can cause both clinical and subclinical disease in infected animals [1]. The environmental route of transmission is important for many gastrointestinal protozoan and helminth parasites, with water, soil, and feed being particularly significant [1]. *Eimeria* and *Cryptosporidia* are among the most common protozoan parasites in calves, and they have been associated with diarrhea, causing decreased growth rates and mortalities, and hence adversely affecting the dairy enterprise [2,3].

Bovine eimeriosis is a common worldwide parasitic infection, causing great economic losses [4]. The condition is observed in animals of all ages, but younger animals are more affected than adults [5-7] in part due to the undeveloped immunity of newborn calves. *Eimeria* infections are more common in confined animals than those on pastures due to the build-up of the oocysts in the environment of confined animals with poor hygiene in the calf pens [8]. Inadequate nutrition and overcrowding increases prevalence of the disease due to the immunosuppression they cause [8]. Animals feeding directly on the ground or grazing on pasture are also more likely to ingest oocysts than those fed in feed troughs [8]. Moreover, the disease is more common in the wet season than in the dry season [4,5], and female animals have been observed to be more susceptible than males [9].

*Cryptosporidia* infection causes a number of economic losses to the dairy industry, among them are mortalities, retarded growth of the animals, cost of drugs, and veterinary assistance and increased labor [9]. Infection by the parasite leads to variable degrees of shedding of the oocysts, where initial shedding can start long before diarrhea starts and continues long after diarrhea ceases [9]. Calves between 1 and 2 months of age have been found to have a higher prevalence (42%) compared to those less than a month old (28%), and those older than 3 months (30%) [10]. However, other studies [9] reported a higher prevalence in calves <1 month than in older ages. Females have been reported to have a higher prevalence than males [9] although no sex preponderance was observed in another study [9]. The high incidence in calves has been associated with poor immunity in newborn calves and bucket feeding with inappropriate hygiene [9]. Dirty calf pens have been associated with high risk of the disease, and very low doses of the oocysts can cause the disease [10].

With division of land among children, smallholder dairy farmers in Kenya now frequently live on small plots of land where calves and cows are raised in close proximity in small zero-grazing units, potentially increasing the transmission risks for these protozoan gastrointestinal infections. It is unclear whether the above, or other, risk factors contribute to the protozoan infections on smallholder dairy farms in Kenya.

This study was, therefore, undertaken to determine the risk factors associated with occurrence of *Eimeria*, *Cryptosporidia*, and diarrhea in calves among smallholder dairy farms in Kenya. This information will enable farmers in this area, and areas with similar management, to put in place appropriate control measures for these protozoan infections.

## Materials and Methods

### Ethical approval

The study was approved by Biosecurity, Animal use and Ethics Committee, Faculty of Veterinary Medicine, University of Nairobi. The farmers who were recruited into the study signed a consent form.

### Study area

The study was undertaken in Mukurwe-ini Sub-County of Nyeri County. Nyeri County is one of the counties of the former Central Province; the other counties include Kiambu, Kirinyaga, Muranga, and Nyandarua. Nyeri County is located between longitude 36° and 38° east and between the equator and latitude 1° south. Mount Kenya is located to the east of Nyeri County at an altitude of 5199 m, and the Aberdare Range is to the west at 3999 m [11]. The study area is at an altitude range of 1500 and 2500 m, and where there is annual rainfall of over 1000 mm and humidity >50% [19], making it suitable for coffee, tea, and dairy production [12].

### Farm selection

Dairy farms used in this study were selected from Mukurwe-ini Wakulima Dairy Limited (MWDL), which has approximately 6000 active members. Purposive sampling was used to select smallholder farms (i.e., having up to 4 cows) to be included in the study. Artificial insemination records kept by the dairy company were queried to identify farms with dams that were due to calf in the months of June and July 2013. The farms whose records indicated that they had a cow that was to calve within the study period were contacted by phone and invited to participate in the study. To be included, farmers had to agree to keep the calf for at least the first 8 weeks of its life (the study period). When a farmer agreed to participate in the study, he/she was requested to contact MWDL as soon as the cow calved. A total of 111 farms were initially eligible, selected, and agreed to participate in the study, with a total number of 112 calves (one farm had twins).

### Data and sample collection

A questionnaire was used to collect data on calf, farm, and household characteristics. Before being applied to this study, the questionnaire was pre-tested through a pilot study in the same area to allow for consistency in understanding and administration of the questionnaire questions. The study questions were answered by the principal farmer, the spouse, or the employees involved in primary animal care, depending on the person available. The questionnaire was administered in English and translated into the local language (Kikuyu) or Kiswahili, depending on the level of farmers’ understanding of the language. The responses were then recorded in English. The first section of the questionnaire (farm and household characteristics) was administered on the first visit to the farm (within 7 days of birth of the study calf).

On subsequent visits, which were done every 2 weeks, the second section of the questionnaire was administered to collect data from the farmer on the calf’s feeding and health during the previous 2 weeks. A physical examination was also performed to assess the calf’s health. This involved taking a rectal temperature, auscultation of the lungs and heart, palpation of the navel, observation of mucous membranes, hair coat, soiling of perineal area, and assessment of hydration level. Girth weights of the calves were also taken during each visit for determination of average weight gains. In addition, assessment of comfort of the calf and hygiene of the pens was done at each visit. Comfort was assessed as a function of space, shade, and softness of the floor of the calf pen (poor vs. good), whereas hygiene was assessed as a function of bedding dryness, manure accumulation, and calf soiling (poor vs. good). The conditions had to be acceptable on at least 2 of 3 items to qualify as good calf comfort or good calf hygiene.

Two fecal samples were collected per rectum from each calf; one at 4 weeks and the second one at 6 weeks of the study period. The samples were placed in fecal bottles, labeled, and transported in a cool box to the laboratory for analysis. A total of 220 fecal samples (110 at 4 weeks and 110 at 6 weeks) were collected (2 calves died before the 4-week visit).

During the first visit, blood samples were collected from the jugular vein into red-top plain vacutainer^®^ tubes, which were then placed in an upright position in a cool box until clotting occurred and clear serum was seen above the clot - this was after several hours. The sample was then centrifuged at 3000 revolutions per minute for 5 min, and the serum was collected and transferred into cryovials for storage at −20°C in the biochemistry laboratory at the University of Nairobi until all samples were collected.

### Sample processing and laboratory analysis

The total serum protein and selenium levels were used to assess the level of passive transfer of immunity to the calves [13]. Selenium has been demonstrated as an important mineral in the development of calves’ immune systems (improvement of bactericidal activity of neutrophil granulocytes, and increase in antibody production) [13]. Selenium analysis was done using an AAS-990 spectrophotometer. The atomic spectroscopy applied the principle of using higher energy radiation to transit inner electrons from low to high energy states. Flameless graphite furnace system was used as a source of the high energy to nebulize the sample. In the selenium analysis, hydride generation atomic absorption, spectroscopy, was used due to the volatility of selenium. Selenium (Se) values of 0.1 ppm were considered adequate for the study calves while those with lower levels had a deficit.

The Biuret method was used for analysis of total protein. This method has been described to have a high sensitivity in assessing total serum protein concentration [14]. The principle of the test is that when copper ions (reagent) react with proteins (in the sample) in an alkaline solution (reagent), violet-blue results. The absorbance of the color is directly proportional to the concentration of the proteins in the sample, and the results are read in g/dl. Studies have shown that a serum protein concentration of 5.2 g/dl is equivalent to 1000 mg/dL serum immunoglobulin G (IgG), and this is defined as adequate transfer of passive immunity, whereas lower values are an indication of failure of transfer of passive immunity (FTPI) [15].

Sheather’s sucrose floatation method was used to harvest the *Cryptosporidium* oocysts which were subsequently stained using modified Ziehl-Neelsen, as described by Clarke and McIntyre [15]. The McMaster technique was used for determination of the presence of *Eimeria* oocysts in the feces.

### Statistical analysis

The questionnaire (household, calf, and management) and laboratory data were entered into an Excel spreadsheet v2010 (Microsoft, Redmond WA, USA) before being imported to SPSS Statistics v20 for Windows (IBM, Armonk, NY, USA) for statistical analysis.

Descriptive statistical analyses were done to summarize the household, calf, and management factors into frequencies and proportions (dichotomous data) and measures of central tendency and variation (continuous variables). Incidence risk of diarrhea and period prevalence of infections with *Cryptosporidia* and *Eimeria* were calculated.

Univariable logistic regression analysis was used to assess associations between risk factors (household factors and calf management factors) and occurrence of diarrhea, *Cryptosporidia*, and *Eimeria*. Those factors that were significant at p≤0.20 were placed in a multivariable logistic regression model. Backward elimination method was used to drop factors that were not significant so that the final model had only significant and confounding factors. Factors were considered significant in the final model if they had p≤0.05. Seven household factors (gender, marital status, education level of farmer, income contribution by dairy farming, years in Wakulima Dairy Limited [WDL], farm size, and land rented) and 12 calf management factors (supervision of birth, breed of calf, sex of calf, categorized time of birth, assistance of birth, vigor of calf, method of colostrum administration, time of colostrum consumption, average daily amount of milk and pellets consumed pre-weaning, calf pen hygiene, and comfort) were assessed. The prevalence of the parasite infections was also considered a possible risk factor for diarrhea.

Linear regression models were used to assess the effects of parasite infections and diarrhea on the average daily weight gains of the calves (as a measure of impaired growth performance). Initially, univariable linear regression was employed to assess associations between factors (household factors, calf management factors, and prevalence of the parasite infections) and average daily gain. Those factors that were significant at p≤0.20 were put into a multivariable linear regression model. Backward elimination method was used to drop factors that were not significant so that the final model had only significant and confounding factors. Factors were considered significant in the final model if they had p≤0.05.

## Results

### Farm demographics

In this study, 111 smallholder dairy farms were included, and half of them were female headed. More than three quarters of the farmers included in the study were married. Education levels varied among the farmers and even between the two genders, with females having somewhat lower levels of secondary and tertiary education than males (Table-1).

**Table-1:** Household demographics in 111 smallholder dairy farms in Mukurweini district, Kenya, 2013.

Characteristic (factor)	Categories	Frequency	Percentage
Gender	Male	55/111	49.5
	Female	56/111	50.5
Marital status	Married	94/111	84.7
	Widowed or single	17/111	15.3
Education level (men)	Tertiary or secondary	48/99	48.5
	Primary	45/99	45.5
	None	6/99	6.1
Education level (women)	Tertiary or secondary	39/106	36.8
	Primary	56/106	52.8
	None	11/106	10.4
Contribution of dairy farming to household income	>70%	29/109	26.6
	50-70%	38/109	34.9
	<50%	42/109	38.5
Years as a member of MWDL	>10	71/111	64.0
	7-9	15/111	13.5
	4-6	12/111	10.8
	1-3	13/111	11.7

MWDL: Mukurweini Wakulima Dairy Limited

A majority, 64% (71/111), of the farmers have been members of MWDL for more than 10 years. The income contribution of dairy farming to the households interviewed was relatively evenly distributed among the three categories (Table-1). Two farmers did not answer the income question.

The pieces of land farmed in Mukurwe-ini were small, with the smallholder farmers owning an average of 1.88 acres of land, and some farmers owning as little as 0.125 acres. This led to 38.7% (43/111) of the farmers renting and from their neighbors to plant-animal fodder, with some renting up to 8.3 acres of land.

### Calf management practices

One calf from each of the 111 farms was included in the study, except for one farm which had twins. 112 calves were, therefore, included in this study, and their management is summarized in Table-2. Two calves died between the first and second visit to the farms (from birth to 2 weeks of age); therefore, some calf parameters were based on denominators of 110 calves.

**Table-2:** Calf management practices for 112 calves in 111 smallholder dairy farms in Mukurwe-ini Sub-County, Kenya.

Characteristic	Categories	Frequency of responses	Percentage
Breed of calf	Friesian-crosses	108/112	96.4
	Other crosses	4/112	3.6
Supervision of birth	Yes	95/112	84.8
	No	17/112	15.2
Time of birth	Morning	46/112	41.1
	Afternoon	25/112	22.3
	Night	41/112	36.6
Assistance of birth	No	69/112	61.6
	Yes	43/112	38.4
Vigor of newborn calf	Strong	103/112	92.0
	Weak	9/112	8.0
Method of colostrum administration	Bucket	68/112	60.7
	Free suckle	28/112	25.0
	Nursing bottle	10/112	8.9
	Assisted suckle	6/112	5.4
Time when 4 L colostrum was consumed	<6 h	53/112	47.3
	6-12 h	27/112	24.1
	12-24 h	18/112	16.1
	>24 h	14/112	12.5
Average amount of milk fed (L/day)	≤5	61/110	55.5
	>5	48/110	43.6
Average amount of calf pellets fed (g/day)	0-250	31/110	28.2
	251-500	53/110	48.2
	>500	26/110	23.6
Average calf pen hygiene	Good	52/110	47.3
	Poor	58/110	52.7
Average calf pen comfort	Good	37/110	33.6
	Poor	73/110	66.4

Most calves were Friesian crosses, and more than half of the calves were male (63/112). Supervision of birth was done in 84.8% (95/112) of the parturitions, despite the births being widely spread out throughout the day. A majority of the births were unassisted, and most calves were born strong, whereas 8.0% (9/112) were born weak and required assistance, especially supportive breathing.

The majority of farmers fed colostrum to their calves using a bucket, and nearly half of farmers reported having fed at least 4 L of colostrum in <6 h after birth, whereas 12.5% of farmers took more than 24 h to feed 4 L of colostrum.

A majority of the farmers fed their calves more than 5 L of milk/day, but less than half of farmers provided good comfort and hygiene to their calves.

### Calf health

Diarrhea and navel ill were the major conditions experienced by the calves. The two calves lost to follow-up died before they experienced either of the two conditions. The incidence risk of diarrhea was 37.3% with (41/110) calves developing at least 1 case of diarrhea in the 2-month study period. The incidence risk of navel ill reported was 17.3% (19/110).

### Calf parasites, total protein, and selenium levels

The period prevalence of the fecal parasites was 42.7% (47/110) and 13.6% (15/110) for *Eimeria* and *Cryptosporidia*, respectively. Serum total protein and selenium levels are shown in Table-3. Nearly, a third of the calves had low total protein serum levels, whereas only a small number of study calves had low serum selenium levels. There was insufficient serum to conduct serum total protein and selenium assays on 1 and 8 samples, respectively.

**Table-3:** Average daily gain and serum total protein and selenium levels of 112 calves in 110 smallholder dairy farms in Mukurwe-ini Sub-County, Kenya.

Parameter	Mean	Median	Standard deviation	Standard error of mean	Categories	Frequency	Percentage
Average daily weight gain (g)*	602.3	594.6	157.5	15.0	n/a	n/a	n/a
Serum protein levels	6.1	5.8	1.8	0.17	High protein (>5.2 g/dl) Low protein (<5.2 g/dl)	78/111 33/111	70.3 29.7
Serum selenium levels	1.03	0.60	1.84	0.18	High selenium (>0.1 ppm) Low selenium (<0.1 ppm)	101/104 3 (104)	97.1 2.9

* Only 110 calves on 109 farms because 2 calves died during first 2 weeks of life

### Associations between infections and farm, calf and management factors

#### Factors associated with occurrence of Eimeria infections

Calf vigor (p=0.19), serum protein levels (p=0.02), and time when 4 L of colostrum was consumed by the calf (p=0.04) were factors that met the univariable regression cutoff p=0.20 for modeling associated with *Eimeria* infection. When these factors were modeled in multivariable regression models, only serum protein level (p=0.02) remained significantly associated with *Eimeria* infection. Calves with low serum protein levels (<5.2 g/dl) had 1.8 (95% confidence interval [CI]: 1.6-1.9) times higher odds of *Eimeria* infection than those with high serum protein level.

#### Factors associated with occurrence of cryptosporidia infection

Serum protein levels (p=0.01), daily average amount of pellets consumed (p=0.11), daily average amount of milk consumed (p=0.05), and average calf pen hygiene (p=0.08) were factors that met the univariable regression cutoff p=0.20 for modeling associated with *Cryptosporidia* infection. In the multivariable modeling process, serum protein level (p=0.01) was the only factor that remained significantly associated with *Cryptosporidia*. The odds of *Cryptosporidia* in calves with low serum protein (<5.2 g/dl) were 2.4 (95% CI: 1.3-4.7) times higher than those with high serum protein.

#### Factors associated with incidence of diarrhea

Duration of years in the WDL (p=0.01), supervision of birth (p=0.02), time of birth (p=0.05), and serum protein levels (p=0.01) were factors that met the univariable regression cutoff p=0.20 for modeling associated with incidence risk of diarrhea. In the final multivariable model, duration of years at Wakulima Dairy, supervision of birth, and serum protein levels remained significant (Table-4). The odds of diarrhea in calves from farmers who had been WDL members for 7-9 years were 0.6 (95% CI: 0.5-0.8) times the odds in calves from farmers who had been members 10 or more years. There was, however, no significant difference in the incidence of calf diarrhea among farmers who had been members of WDL for less years (<7 years) compared to those who had been members for 10 or more years (referent category). The odds of diarrhea were 1.3 (95% CI: 1.0-1.6) times higher when the birth of the calf was not observed than when it was observed. Calves with low serum protein (<5.2 g/dl) had 1.3 (95% CI: 1.1-1.6) times higher odds of diarrhea than those with high serum protein levels. The diagnosis of *Eimeria* and *Cryptosporidia* infections was not associated with diarrhea (p>0.05). There were no significant interactions among the significant model variables.

**Table-4:** Multivariable logistic regression results of factors associated with diarrhea incidence in 110 calves reared in 109 smallholder dairy farms in Mukurwe-ini Sub-County, Kenya.

Parameter	p value (factor)	Categories	Chisquare	Odds ratio	p value (categories)
Duration of years in WDL (reference ≥10 years)	0.003	1-3	1.20	0.87	0.273
		4-6	0.23	0.94	0.633
		7-9	12.33	0.64	<0.001
Birth supervision (reference=yes)	0.023	No	5.80	1.32	N/A
Serum protein levels (reference=high)	0.004	Low	8.52	1.34	N/A

WDL: Wakulima Dairy Limited

#### Factors associated with average daily weight gain

The overall average daily weight gain in the calves was 0.60 kg/day (95% CI: 0.57 to 0.63 kg/day). Gender of the principal farmer (p=0.19), marital status of the principal farmer (p=0.18), vigor of the calf at birth (p=0.15), average daily milk consumed (p<0.001), calf pen hygiene (p=0.04), and *Eimeria* infection (p=0.19) were factors that met the univariable regression cutoff p=0.20 for modeling associated with average daily gain. In the multivariable linear regression model, calf pen hygiene, *Eimeria* infection, and average milk consumed were the factors that remained significantly associated with average daily weight gain of the calves (Table-5). Calves that were kept in pens with good hygiene had an average daily weight gain of 0.63 kg/day (95% CI: 0.59-0.67 kg/day), whereas for those kept in dirty calf pens, the average weight gain was 0.57 kg/day (95% CI: 0.53-0.61 kg/day). Calves that were fed 5 or more liters of milk per day had an average daily weight gain of 0.65 kg/day (95% CI: 0.60-0.69 kg/day) compared to the 0.56 kg/day (95% CI: 0.52-0.59 kg/day) found among calves getting <5 L/day. Calves that were infected with *Eimeria* at least once within the 2 months of study had an average daily weight gain of 0.58 kg/day (95% CI: 0.53-0.62 kg/day) while those who were not infected had average weight gain of 0.62 kg/day (95% CI: 0.57-0.66 kg/day). There were no significant interactions among the significant model variables.

**Table-5:** Multivariable linear regression results of factors associated with average daily weight gain of 110 calves reared in 109 smallholder farms in Mukurwe-ini Sub-County, Kenya.

Parameter	Categories	Estimates	p value
Average daily milk consumed (liters) (reference <5)	≥5	0.1050	<0.001
Average calf pen hygiene (reference=poor)	Good	0.0868	0.003
*Eimeria* infection (reference=negative)	Positive	−0.0591	0.043

## Discussion

In the Kenyan context, the results of this study suggest that calf diarrhea risk could be reduced not only through proper colostrum provision for optimizing passive transfer of immunity to the newborn calves but also through better supervision of parturition, which likely led to reduced calf stress. Producers should also be informed that good pen hygiene leads to higher average weight gains, likely through reduced exposure to fecal pathogens, particularly *Eimeria*.

Time, quantity, and quality of colostrum consumed by a newborn calf are key factors in increasing passive immunity, and hence, the rearing of healthy calves [13,16]. Ingestion of at least 3-4 L of quality colostrum within the first 1-2 h of birth has been shown to decrease the incidence of failure of passive transfer of immunity in newborn calves [17]. FTPI is known to predispose calves to the development of neonatal diseases [17,18]. *Eimeria* and *Cryptosporidia* infections are some of the neonatal diseases which occur, in part, as a consequence of inadequate colostral immunity [13].

Low serum protein resulted in increased incidence risk of diarrhea in our study calves. Because of the syndesmochorial placenta of the ruminant, the calf is born with low levels of gamma globulins, thereby depending on colostrum for adequate Igs (and immunity) [17,18]. Measurement of serum total protein as an estimate of serum IgG concentration has been used as an effective method of determining passive transfer of immunity in calves [18]. Within the 1^st^ week after birth, a strong correlation (0.71) between total protein and IgG has been observed, and this has been used as a measure of immunity obtained by the calves [17]. Hence, a low serum total protein level of a calf is an indication of low Igs in the serum and reduced immunity. Failure of passive transfer is known to increase mortality and morbidity of calves [18].

Contrary to findings elsewhere [19], for calves up to 2 months of age, we found no association between the occurrence of *Cryptosporidia* infections and diarrhea. This study’s findings, however, agree with Ayinmode and Fagbemi study’s findings [20], who reported that shedding of *Cryptosporidia* oocysts is not always associated with diarrhea. Diarrhea may have been from the presence of other diarrhea-causing enteropathogens such as rotavirus or *Escherichia coli* infections [21]. The occurrence of *Cryptosporidia* in asymptomatic calves may indicate this group of animals as the reservoirs for this parasite [13]. The absence of an association between *Cryptosporidia* shedding and diarrhea may also be because of the low number of infections found in this study. The prolonged period of oocyte shedding long before onset and after cessation of diarrhea [13] may increase cases of subclinical cryptosporidiosis, as seen in our study.

During the study period, diarrhea was reported at least once in 37.3% of the calves. Calf diarrhea is a major challenge in the smallholder dairy sector, as reported in different regions; 27% in Kenya [22] and 73% in Canada [23]. Diarrhea incidence risk was, however, not associated with the occurrence of *Eimeria* and *Cryptosporidia* infections between calf diarrhea and diarrheic agents that they investigated such as *Salmonella*, *Campylobacter*, and *E. coli*. Baumgartner [24] reported that in a majority of calf diarrhea cases, more than one enteropathogen is usually involved; hence, there is a possibility of masking the effects of either *Eimeria* or *Cryptosporidia* as the causes of diarrhea. Other possible reasons for the lack of association include: Shedding of the parasite that did not coincide with the sampling occasion; failure to detect the causative parasite; non-infectious causes of diarrhea (e.g., nutritional), and non-parasitic infectious causes of diarrhea (e.g., viral or bacterial). Diarrhea in smallholder dairy farms has been associated with the improper feeding of the calves, especially overfeeding of milk [25].

Lack of calving supervision also resulted in increased cases of diarrhea. Good supervision of calving involves being present to offer necessary assistance during stage two of labor or to call for veterinary assistance if required [26] and to ensure colostrum consumption soon after birth. Early human intervention after calving has been reported to influence calf health since the calf is born from a sterile environment to one with a large variety of potential pathogens [13]. Lack of calving supervision has been reported to cause calf morbidities and/or mortalities through anoxia following dystocia and acidosis, which may lead to failure of transfer of maternal Igs [26], hence increasing the risk of infection. In addition, lack of supervision during parturition may result in the calf not being moved quickly from a contaminated environment; the calf may then start to nibble the manure-stained bedding in an effort to find something to eat, and this may lead to increased diarrhea [13].

Calves from farms which have been members of WDL for between 7 and 9 years had less cases of diarrhea than those who had been members of the dairy for more than 10 years. There was, however, no significant difference between cases of calf diarrhea experienced by farmers who had been members of WDL for less years (<7 years) and those who had been members for 10 or more years. Despite the fact that farmers who had been members for between 7 and 9 years and those who had been members for 10 or more years had gained considerable experience in calf management, the latter may have been less adherent to calf diarrhea preventive measures than their counterparts.

Mean average daily weight gain was 0.60 kg/day. This gain is within the ideal and recommended growth rates in calves, which ranges between 0.5 and 0.73 kg/day for dairy breeds [27]. This ideal average daily weight gain was, however, influenced by supplementation of some calves with additional calf pellets and milk as part of a related feeding trial.

Calf pen hygiene, feeding of more than 5 L of milk/day, and infection with *Eimeria* were associated with average daily weight gain of the calves. Calves in clean calf pens had greater average daily weight gains than those kept in dirty calf pens. Microclimate of farm premises has been reported to have a direct influence on the feeding, health and, consequently, productivity of animals [28]. Calves in clean pens tend to improve feed intake and hence average daily weight gains [29].

In our study, higher milk intake (>5 L/day) by the calves was shown to increase average daily weight gains. Optimum calf nutrition, including feeding enough milk to the calves, has been shown to increase calf survival, improves health and immunity [30], improves future milk production of heifer calves [31], and optimizes calf growth [31]. Calves fed more milk (>5 L/day) had growth rates of 0.65 kg/day compared to 0.56 kg/day for those fed less milk (<5 L/day). Faster growth rates have been reported when milk intake in calves are increased above-maintenance levels [13].

Our study showed a decrease in average daily weight gain in the calves that were infected with *Eimeria*. Bovine eimeriosis has been reported to cause decreased growth rates, especially in young calves [3,4]. During the multiplication of the merozoites, damage to the microvilli leads to impaired absorption of nutrients [11]. The disease also causes decreased feed consumption and feed efficiency, hence resulting in decreased weight gain of the affected animals [4]. Most *Eimeria* infections, however, are subclinical [4,32], and this was the case in our study where no calves showed clinical signs of *Eimeria* infection when positive samples were taken, although some owners reported a history of diarrhea.

The sampling of this study was not random because farms needed to be purposively selected to ensure that they had a newborn calf. However, we feel that the study population is representative of the general population of smallholder dairy farmers in Kenya, for the following reasons. The number of female-headed dairy households was similar to the number of male-headed households, where 50% of smallholder dairy farms in Nyandarua were female headed [33]. The 112 calves involved in the study were mostly Friesian crossbreds, which is characteristic of dairy animals kept in the Kenyan highlands [22].

## Conclusion

Low serum total protein concentration in the calves, an indication of low passive immunity, increased the risk of diarrhea, and infection with *Cryptosporidia* and *Eimeria*. There is, therefore, need to optimize transfer of passive immunity to newborn calves. Lack of supervision of calving was also associated with increased occurrence of diarrhea. *Eimeria* infection, poor calf pen hygiene, and low milk consumption were associated with reduced average daily weight gain of the calves. Profitability of a dairy farm depends on faster growth rates of calves as replacement stock. It is, therefore, important to reduce infection of calves with *Eimeria* (in addition to other neonatal diseases) through proper colostrum consumption, increase milk feeding beyond 5 L/day, and ensure clean calf pens. In combination, these management factors will contribute to increased average weight gain and productivity of the dairy stock.

Future research on other specific causes of calf diarrhea in this area is important to develop improved intervention strategies. Moreover, identification and assessment of management factors that are potential risk factors for cryptosporidiosis among people on smallholder dairy farms are needed. Training strategies for improving colostrum management for farmers, especially timing of ingestion, should also be examined to enhance transfer of maternal immunity to the calf, hence ensuring good calf health.

## Authors’ Contributions

SGP, SR, and JAV conducted the field data collection. SGP and RRK were involved in laboratory sample analysis. SGP, GKG, and JAV wrote the draft manuscript. All authors were involved in the preparation of data collection materials, and the revision and approval of the final manuscript. JAV, GKG, FU, and JAV were involved in funding acquisition. All authors read and approved the final manuscript.
